# *Staphylococcus aureus* Bacteremia, Australia

**DOI:** 10.3201/eid1104.040772

**Published:** 2005-04

**Authors:** Peter Collignon, Graeme R. Nimmo, Thomas Gottlieb, Iain B. Gosbell, on behalf of the Australian Group on Antimicrobial Resistance

**Affiliations:** * The Canberra Hospital, Garran, Australian Capital Territory, Australia;; † Queensland Health Pathology Service, Brisbane, Queensland, Australia;; ‡ Concord Hospital, Concord, New South Wales, Australia;; § Southwestern Area Pathology Service, Liverpool, New South Wales, Australia

**Keywords:** Staphylococcus aureus, bacteremia, hospital infections, methicillin resistance, mortality, fatal outcome, nosocomial infections, infection control, indwelling catheters

## Abstract

*Staphylococcus aureus* bacteremia (SAB) is common and increasing worldwide. A retrospective review was undertaken to quantify the number of cases, their place of acquisition, and the proportions caused by methicillin-resistant *S. aureus* (MRSA) in 17 hospitals in Australia. Of 3,192 episodes, 1,571 (49%) were community onset. MRSA caused 40% of hospital-onset episodes and 12% of community-onset episodes. The median rate of SAB was 1.48/1,000 admissions (range 0.61–3.24; median rate for hospital-onset SAB was 0.7/1,000 and for community onset 0.8/1,000 admissions). Using these rates, we estimate that ≈6,900 episodes of SAB occur annually in Australia (35/100,000 population). SAB is common, and a substantial proportion of cases may be preventable. The epidemiology is evolving, with >10% of community-onset SAB now caused by MRSA. This is an emerging infectious disease concern and is likely to impact on empiric antimicrobial drug prescribing in suspected cases of SAB.

## Abstract 2

*S. aureus* bacteremia in Australia is increasingly caused by MRSA, which is likely to affect empiric prescribing of antimicrobial drugs in suspected cases.

This page was updated on August 30, 2005 to incorporate the corrections in Vol. 11, No. 10.

Bacteremia caused by *Staphylococcus aureus* continues to be a common problem worldwide. In the preantibiotic era, most cases occurred in young patients without underlying disease. The associated death rate was 82% (1). Even with antimicrobial drug treatment, death rates remain high; in a recent meta-analysis of 31 studies, estimates of death rates for methicillin-resistant strains (MRSA) varied from 0.0% to 83.3% (median 34.2%), while those for methicillin-sensitive strains (MSSA) varied from 3.6% to 51.7% (median 25.0%) (2). Many of these infections are healthcare associated and thus are potentially preventable.

Antimicrobial drug resistance in *S. aureus* arose early after the development of antimicrobial agents and continues to evolve. In Australia, hospital strains are frequently methicillin resistant and resistant to several other antimicrobial drugs (3). This resistance limits the choice of potentially efficacious agents and results in frequent use of glycopeptides, such as vancomycin. The reliance on vancomycin causes difficulties because vancomycin has been shown to be less effective than isoxazolyl penicillins (e.g., flucloxacillin) in treating severe infections caused by *S. aureus* (4,5). This may be 1 explanation for the higher death rate associated with bacteremia caused by MRSA, compared with that caused by MSSA (2,6). Although MRSA tends to be the bacterium discussed most often in relation to healthcare-associated infections, MSSA strains are responsible for the largest proportion of hospital-acquired infections (3).

*S. aureus* remains a common cause of bloodstream infections of community onset. Increasing numbers of these community-onset infections are being caused by MRSA. Some of these infections may be caused by hospital strains carried into the community by patients or healthcare workers, but others are caused by true community strains in patients who have had no recent healthcare contact (7–9). These strains have emerged in many countries, including Australia, New Zealand, the United States, Canada, France, Switzerland, Greece, Denmark, Finland, Scotland, and the Netherlands. They are susceptible to most or all non–β-lactam antimicrobial drugs, are highly pyogenic, and are often associated with indigenous populations (10,11).

Although *S. aureus* is a well-known major cause of bacteremia, population-based estimates of its incidence are lacking. This study used hospital data to estimate the incidence of *S. aureus* bacteremia in Australia. In addition, we classified episodes on the basis of community or hospital onset and on the basis of methicillin susceptibility.

## Methods

*S. aureus* bacteremia data were obtained from microbiology departments that prospectively collected information for >12 months on episodes of laboratory-confirmed bacteremia for the hospitals they serviced from January 1, 1999, to December 31, 2002. Information retrieved from existing databases included the total number of episodes of community- and hospital-onset bacteremia, the number of episodes of community- and hospital-onset MRSA and MSSA bacteremia, the total number of hospital separations (defined as completed hospital admissions), and the mean length of stay. Multiple positive blood cultures in the same patient within 14 days were considered a single episode. Episodes were considered to have a hospital onset when the first positive blood culture was collected >48 hours after admission to hospital. All other infections were designated community onset (for example, day-only dialysis related episodes were defined as community onset, as were infections with their onset in nursing homes). Organism identification and susceptibility testing were by standard methods. All these laboratories participate in external quality assurance programs as well as AGAR national surveys (3,12), which have quality control procedures to ensure these laboratories accurately detect methicillin resistance. Published data were used for the details on the number of hospital beds and separations for Australia and for the classification of different types of hospitals (13). The term separations, rather than admissions, is used in the published data because hospital abstracts for inpatient care are based on information gathered at the time of discharge. We have used the more commonly applied term of admissions, however, for these episodes.

In Australia, most healthcare-associated MRSA is caused by 1 clone defined by multilocus sequence type (ST) 239; this clone is characteristically resistant to multiple antimicrobial agents, including gentamicin (3,12). Most of the remaining healthcare-associated infections are caused by a recently introduced strain, ST22, which is indistinguishable from epidemic MRSA-15 in the United Kingdom. It is invariably resistant to ciprofloxacin (12). Thus, in Australia, MRSA that is acquired in the community and is sensitive to both ciprofloxacin and gentamicin is not likely to be associated with healthcare facility acquisition. We used this pattern as a surrogate marker for community acquisition of MRSA without healthcare-associated risk factors.

## Results

We detected 12,771 bloodstream infections in the 17 hospitals participating in this study (12 principal referral metropolitan, 3 large metropolitan, 1 private hospital, and 1 medium-sized public hospital, and 1 private hospital with 2,013,534 total separations; Table 1). There were 3,192 episodes of *S. aureus* bacteremia identified (i.e., 25% of the total true bloodstream infections). The median rate of *S. aureus* bacteremia was higher in the principal referral metropolitan hospitals (1.59/1000 admissions) than in large metropolitan hospitals (1.3) or the private hospital (0.6). The range varied from 0.60 to 3.24 (Table 2). The median rate of community-onset bacteremia episodes was 0.80/1000 admissions (range 0.11–0.99). The median rate of hospital-onset bacteremia was 0.72 episodes/1,000 admissions (range 0.13–1.30). The median rate of hospital-onset MRSA episodes was 0.22/1,000 admissions (range 0–0.89). When expressed as MRSA episodes per 1,000 occupied bed days (OBDs), the rates varied from 0 to 0.30 with a median rate of 0.08. If day-only cases are removed from the denominator then the median rate was 0.10 per 1,000 OBDs (range 0–0.39).

**Table 1:** Bacteremia episodes at individual hospitals*

	Hospital								
	A	B	C	D	E	F	G	H	I
Classification†	a	a	a	a	a	a	a	a	a
Beds	723	587	551	525	504	468	455	394	391
Years studied	4‡	4‡	3§	4‡	4‡	3§	4‡	4‡	2¶
Admissions over study period	256,251	203,130	150,502	204,116	194,246	132,781	185,680	175,583	67,855
Admissions >24 h over study period	66,035	76,147	49,501	102,361	60,498	42,515	39,758	44,502	47,406
Mean length of stay (day cases included)	3.85	3.48	3.84	3.6	3.43	3.8	3.61	3.32	4.25
OBDs (including day-only patients)	986,566	706,892	577,928	781,235	666,264	504,568	670,305	582,936	288,384
OBDs (excluding day-only patients)	796,350	579,909	476,927	679,481	532,516	414,302	524,383	451,855	267,935
Total *S. aureus* bacteremia	331	365	333	373	267	107	426	259	115
Total BSIs over study period (all orgs)	1,531	1,172	1,294	1,546	1,296	605	1,689	1,120	472
Total BSI rate per hosp admissions (x1,000)	5.97	5.76	8.60	7.57	6.67	4.50	9.01	6.38	6.95
	Hospital								
	J	K	L	M	N	O	P	Q	Total
Classification†	a	a	a	b	b	b	c	d	–
Beds	368	297	276	199	170	162	72	52	6,194
Years studied	4‡	2¶	4‡	4‡	4‡	4‡	4‡	4‡	–
Admissions over study period	104,534	58,549	92,114	64,311	41,690	48,900	18,223	15,069	2,013,534
Admissions >24 h over study period	50,018	25,617	36,322	31,259	10,556	31,681	13,055	2,894	730,125
Mean length of stay (day cases included)	4.1	4.49	3.06	3.37	5.4	3.60	5.10	2.88	–
OBDs (including day-only patients)	428,589	262,592	281,869	216,728	225,126	176,040	92,937	43,399	7,491,240
OBDs (excluding day-only patients)	374,073	229,660	226,077	183,676	192,874	158,821	87,769	31,224	6,207,832
Total *S. aureus* bacteremia	155	123	72	44	135	62	11	14	3,192
Total BSIs over study period (all orgs)	653	338	351	282	881	274	67	63	12,771
Total BSI rate per hosp admissions (x1,000)	6.25	5.77	3.81	4.39	21.13	5.60	3.68	4.18	–

*MSSA, methicillin-susceptible *Staphylococcus aureus*; MRSA, methicillin-resistant *S. aureus*; OBDs, occupied bed days; BSI, bloodstream infection; orgs, microorganisms. †Hospital classification: a, principal referral: metropolitan (>20,000 acute weighted separations per year) and rural (>16,000 acute weighted separations); b, large metropolitan (>10,000 acute weighted separations); c, private hospital; d, medium sized (metropolitan and rural 2,000 acute or acute weighted to 5,000 acute weighted separations). ‡1999–2002. §1999–2001. ¶1999–2000.

**Table 2:** Rates of *Staphylococcus aureus* bacteremia (SAB) at individual hospitals

	Hospital																
	A	B	C	D	E	F	G	H	I	J	K	L	M	N	O	P	Q
Total *S. aureus* bacteremia (SAB)	331	365	333	373	267	107	426	259	115	155	123	72	44	135	62	11	14
Rate/hospital admissions (x1,000)	1.29	1.80	2.21	1.83	1.37	0.80	2.29	1.48	1.69	1.48	2.10	0.78	0.68	3.24	1.27	0.60	0.93
Rate of community-onset infections*	0.51	0.74	0.94	0.99	0.66	0.35	0.99	0.83	0.77	0.88	1.06	0.58	0.48	2.40	0.80	0.11	0.80
Rate of hospital-onset *S. aureus* infection*	0.78	1.05	1.27	0.84	0.72	0.46	1.30	0.·65	0.93	0.60	1.04	0.21	0.20	0.84	0.47	0.49	0.13
Rate of hospital-onset MSSA *	0.54	0.58	0.74	0.64	0.46	0.42	0.41	0.55	0.49	0.25	0.59	0.10	0.08	0.62	0.41	0.44	0.13
Rate of hospital-onset MRSA*	0.24	0.48	0.53	0.20	0.26	0.04	0.89	0.10	0.44	0.35	0.44	0.11	0.12	0.22	0.06	0·05	0·00
Rate of *S. aureus* SAB sepsis/1000 OBDs	0.34	0.52	0.58	0.48	0.40	0.21	0.64	0.44	0.40	0.36	0.47	0.26	0.20	0.60	0.35	0.12	0.32
MRSA SAB rate/1,000 OBDs	0.08	0.17	0.18	0.08	0.09	0.01	0.30	0.05	0.14	0.15	0.13	0.05	0.05	0.06	0.02	0.01	0.00
MRSA SAB rate/1,000 OBDs- excluding 1 day only	0.10	0.21	0.22	0.09	0.11	0.01	0.39	0.06	0.15	0.17	0.15	0.06	0.05	0.07	0.03	0.01	0.00

*SAB per hospital admissions (x1,000). †OBDs, occupied bed days; MSSA, methicillin-susceptible *S. aureus*; MRSA, methicillin-resistant *S. aureus*.

Of these 3,192 SAB episodes, 1,621 (51%) were of hospital onset, and 1,571 (49%) had their onset in the community. Of those with a hospital onset, 40% were MRSA in comparison to 12% with a community onset. Of all MRSA bacteremia episodes, 23% had a community onset, and 77% had hospital onset. Of the 193 community-onset episodes of MRSA that occurred, only 47 (24%) had a sensitivity pattern (sensitive to gentamicin and ciprofloxacin) that suggests that they were community acquired.

When both MRSA and MSSA were considered, data were available for 560 community-onset SAB infections (but only from 4 hospitals). The proportions of these episodes that were noninpatient, healthcare-associated were 35%, 42%, 18% and 16%, respectively (from hospitals A, D, E, and N). In those hospitals, the percentage of *S. aureus* episodes that were healthcare associated overall (i.e., all hospital-onset cases and those community-onset cases associated with healthcare exposure) were 75%, 69%, 64%, and 36%, respectively.

Mortality data were available for 526 patients from 2 hospitals. At hospital E, the mortality rate at day 7 was 10% (27 of 267 patients). When a subgroup of these patients at hospital E (52 patients) was followed for a longer period (2001–2002), the mortality rate was 23% at 30 days and 35% at 6 months. For those 24 patients with a community-onset episode of bacteremia that was not healthcare associated, mortality rates were 6% at day 7, 17% at 1 month, and 21% at 6 months, respectively. At hospital H (259 patients), the mortality rate at 30 days was 19%. At hospital H, the mean length of stay for those with SAB was 25.6 days compared to 6.2 days in matched controls. The mean length of stay was longer for MRSA infections (39.2 days) than for MSSA infections (23.3 days).

The rates of *S. aureus* bacteremia in different hospital populations were used to estimate the incidence for Australia. Using our median bacteremia rate for *S. aureus* bacteremia in different types of public hospitals (1.27/1,000 admissions, range 0.68–3.24) and in private hospitals (0.6/1,000 admissions), we estimated ≈6,900 episodes per year nationally (range 3,826–20,658) or 35/100,000 per year (Tables 3 and 4). Some data are available from other countries for comparison; the lowest annual rates are in Northern Ireland (23/100,000) and the highest in the United States (56/100,000; Table 4). However only 2 countries, Denmark and England, appeared to have comprehensive collection systems, and their rates were 29/100,000 and 37/100,000, respectively (17,20,22).

**Table 3:** Estimated numbers of Staphylococcus aureus bacteremia, Australia*

	Principal referral public hospitals	Large public hospitals	Medium public hospitals	Small acute-care public hospitals†	Other public hospitals‡	Total acute-care public hospitals§	Total private	Total Australia-wide (based on tally of public and private hospitals)
Published data for Australia 2001–2002 (13)								
Number of hospitals	66	40	103	134	381	724	537¶	1,306
No. of beds	27,258	5,760	6,386	3,216	6,384	49,004	27,407	75,516
Total admissions (x1,000)	2,585	561	486	153	165	3,950	2,426	6,376
Same day separations (x1,000)	–	–	–	–	–	1,886	1,453	–
Average length of stay	3.8	3.6	3.4	–	–	4.1	2.9	3.5
*S. aureus* BSI episodes (calculated rates from data in this study)								
*S. aureus* BSI rate/1,000 admissions	0.81–2.29	0.68–3.24	0.93	0.6	0.6	0.68–3.24	0.6	0.6–3.24
Estimated episodes/y	2,094–5,920	381–1,818	452	92	99	2,370–12,798	1,456	3,826–20,658
Median rate/1,000 admissions	1.59	1.27	0.93	0.6	0.6	1.37	0.6	NA
Estimated episodes/y (based on median)						5,412	1,456	6,867
Hospital-onset MSSA								
Rate/1,000 admissions	0.10–0.74	0.08–0.62	0.13	0.13	0.13	0.08–0.74	0.44	0.10–0.97
Estimated episodes/y	259–1,913	45–347	31	137	21	316–2,923	1,067	638–4,718
Median rate/1,000 admissions	0.51	0.41	0.13	0.13	0	0.47	0.44	NA
Estimated episodes/y (based on median)	1,318	230	63	137	21	1,769	1, 067	2 836
Hospital-onset MRSA								
Rate/1,000 admissions	0.04–0.89	0.06–0.22	0	0	0	0.05–0.89	0.05	0.05–0.89
Estimated episodes/y	103–2,301	34–123	0	0	0	198–3,516	121	255–5,675
Median rate/1,000 admissions	0.31	0.12	0	0	0	0.25	0.05	NA
Estimated episodes/y (based on median)	801	67	0	0	0	868	121	1.015

*BSIs, bloodstream infections; MSSA, methicillin-susceptible *S. aureus*; MRSA, methicillin-resistant *S. aureus*; NA, not applicable. †No data from this study on smaller public hospitals. Therefore the assumed rate of sepsis is for lowest in other groups (i.e., private hospitals). ‡These public hospitals were those without case mix-adjusted admissions data and also non-acute small hospitals. The assumed rate of sepsis is for lowest in other groups (i.e., private hospitals). §Acute care public hospitals exclude psychiatric hospitals. ¶Of private hospitals, 246 were day only; 314 others had admissions for >24 h.

**Table 4:** International rates and numbers of *Staphylococcus aureus* bacteremia (SAB)*

Country	Y	Population	SAB/y	SAB/10^5^/y	% MRSA
Australia					
Present report	1998–2002	19,500,000	6,900	35	27
Victoria (25)†	1990–1999	4,502,000	804	27	28
Denmark					
Northern Jutland (21)	1996–1998	493,000	155	31	ND
Whole of Denmark (17)‡	2002	5,350,000	1,488	28	0.6
Ireland (23)§	1999	3,700,000	ND*	25	36
United Kingdom					
England (20,22)¶	2002–2003 2003	49,200,000	18,403 19,244	37 39	40 41
Northern Ireland (22,24)#	2002 2003	1,697,000	397 569	23 34	38 44
Wales (22)#	2003	2,920,000	742	25	47
USA					
Connecticut (14)**	1998	1,124,337	634	56	ND*

*MRSA, methicillin-resistant *Staphylococcus aureus*; ND, no data given. †In Victoria, 8,036 SAB episodes were reported, resulting in a rate of 17.8/100,000. The final rate (27.0) for the entire state was extrapolated from this figure. The Victorian scheme is estimated to capture about two thirds of all bacteremia episodes that occur in that state per year. ‡System in place in Denmark since 1960, with numbers of episodes continually rising (e.g., in 1966, 400 per year and total population 4.8 million or 8/100,000). Collection data based on reviewing all discharge summaries and laboratory samples (15 of 16 counties). Associated 23% mortality rate in 2002, and 22% of these deaths were directly related to sepsis. §Rates in different regions varied from 8.9 to 37.1 per 100,000. Likely underreporting (22). ¶Compulsory reporting system. Unclear if all community onset episodes were included. In England, underreporting occurred with a voluntary system (only 13,770 episodes reported for 2003; thus, a 50% increase with compulsory system) (22). #This rate is based on voluntary reporting system. Real rate might be 50% higher (22,24). **Retrospective case analysis. Rate increased with age, urban areas, and African American ethnicity. 15% of community-onset SAB episodes were MRSA.

## Discussion

*S. aureus* bacteremia is very common. Approximately one fourth (26%) of all *S. aureus* bacteremia episodes were caused by MRSA, and, as expected, the onset of most of these episodes was in hospitals (77%). Notably, however, 12% of all community-onset *S. aureus* infections were MRSA, which was 23% of all MRSA bloodstream infection episodes. A recent study from the United States similarly showed that 15% of community-onset SAB episodes were MRSA (14). Most of the community-onset strains in our study were multiresistant or phenotypically consistent with UK EMRSA-15 (15) and thus most likely to have been acquired by patients who had previous hospital contact, with nursing home contact a major factor in at least 1 of the hospitals in this study (hospital G). However, approximately one fourth of these community-onset MRSA infections were caused by other phenotypes of non–multiresistant MRSA and thus more likely to be true community-acquired episodes of MRSA bacteremia. Severe cases of MRSA bacteremia not associated with prior healthcare contact have been reported previously in Australia (7,9,16).

Use of the >48 hours postadmission definition of hospital onset underestimates the number of episodes of bacteremia that are healthcare associated. Many patients with chronic conditions are now treated in the community or on a day-only basis. Vascular lines are increasingly used in the community and outpatient settings, providing a potential source of bacteremia. The collection of data on the true association of episodes of bacteremia to health care is time-consuming and was not done by most institutions participating in this study. However, 3 principal referral hospitals (hospitals A, D, and E) did collect these data for 971 episodes, and 64%–75% of their total *S. aureus* bacteremia episodes were healthcare associated. Only 46%–61% of the episodes were acquired while the patient was an inpatient (i.e., >48 h in hospital). This finding means that in these larger hospitals approximately one third of healthcare-associated episodes were acquired by either outpatients or short-stay patients. These episodes are better defined as "noninpatient, healthcare-associated." In a recent study in the United States, 62% of their community-onset SAB infections were healthcare related (with intravenous [IV] catheters the most common clinically apparent site of infection) (14). On the basis of our data, we conclude that in Australia approximately two thirds of all SAB episodes were associated with healthcare or medical procedures (i.e., all hospital-onset and approximately one third of community-onset episodes). A similar situation is evident in Denmark (17), where in 2002, at least 59% of all *S. aureus* infections were associated with healthcare procedures. Clearly, substantial scope exists internationally for interventions in healthcare settings to decrease the numbers of these episodes (especially those related to IV catheters). Interventions to reduce *S. aureus* bacteremia need to target healthcare-associated infections in the broadest sense and include those following non–inpatient-related medical procedures.

Community-onset infections that have no healthcare association are also common and associated with a high death rate (17% and 19% at hospitals E and H at 1 month, respectively). How best to intervene to decrease these infections is difficult to determine. Vaccination is a possibility for the future; a recent trial of a conjugated capsular polysaccharide vaccine in renal dialysis patients estimated efficacy at ≈60% (18). However, vaccination for the general population is unlikely to be available soon. We should therefore concentrate on reducing the number of deaths from established infections. Because the mortality rate associated with community-acquired bacteremia increases with inadequate empiric therapy (19), all efforts should be made to promote compliance with published guidelines for treatment of severe staphylococcal sepsis, including adequate duration of therapy.

Available data suggest that staphylococcal bacteremia is a major global health problem. The median death rate for MSSA infections is 25%, and for MRSA infections, 34% (20). Thus, >1,700 deaths in Australia are likely associated with *S. aureus* bacteremia per year (assuming 6,900 episodes or a bacteremia rate of 35/100,000/year). This estimate of the rate of SAB is similar to England (20,22) but much lower than in the United States on the basis of the rate derived from the figures available in the only comparative study (55/100,000) (14). Our estimated rate in Australia is higher than that in Denmark (17,21). It is also higher that those reported from Wales (22) and Ireland (23) (Table 4); however, all episodes from these last 2 countries likely were not reported in their voluntary reporting schemes. England changed recently from a similar voluntary reporting scheme to a compulsory scheme, and the numbers of reported episodes increased by almost 50% (24).

The rate of MRSA bacteremia in England was higher per 1,000 OBDs than in our figures from Australia (0.17 compared to 0.10 episodes per 1,000 OBDs, respectively). MRSA was a substantial cause of episodes of SAB in this study (26%). However, this percentage was lower than that seen in most other countries (e.g., Wales, 47%; Table 4) with the notable exception of Denmark (0.6% in 2002) (17).

We may have overestimated the number of cases of bacteremia occurring in Australia because of the overrepresentation of larger hospitals in our survey. However, these hospitals participated because they had in place surveillance systems for measuring all episodes of bacteremia. The rates of SAB may have been relatively lower in these hospitals because they were also more likely than were hospitals without surveillance systems to have infection control programs in place to try to decrease the numbers of these episodes. If systems were in place that better captured and reported on all bacteremia episodes in well-defined populations (e.g., all of Australia or a state), then this would give a more accurate rate. Such systems appear only to be in place in Denmark and England (17,21,24). Currently, no such systems are operating in Australia. Limited data are available from a voluntary surveillance system in Victoria (25) that captures an estimated two thirds of bacteremic episodes that occur in that state. The extrapolated rate (27 episodes/100,000 persons/year; Table 4) was slightly lower than what we estimated for all of Australia in this study.

Substantial illness and increased medical costs are also associated with staphylococcal bacteremia. *S. aureus* bacteremia is often related to serious infections, including endocarditis, osteomyelitis, and septic arthritis. It frequently results in prolonged hospital admission and increased costs. In hospital H, the average length of stay for patients with *S. aureus* bacteremia was 26.5 days. In South Australia, the estimated additional cost of each episode of hospital-acquired *S. aureus* infection was $22,000 in 1998 (26). Nationally, these South Australian costs translate to additional hospital costs of ≈$150 million dollars ($22,000 x 6,900 episodes).

Treatment of *S. aureus* infections is complicated by the high prevalence of antimicrobial drug resistance. Although this has long been the case with multiresistant strains of MRSA in hospitals, the spread of hospital strains into the community, as well as the emergence of unique strains of MRSA unrelated to health care, have made this an issue of general importance. At least 3 community strains of MRSA are currently circulating in Australia (10,27,28). Two of these 3 community strains carry the gene for Panton-Valentine leukocidin, which is associated with subcutaneous abscess formation and necrotizing pneumonia. A number of reports have already highlighted the clinical impact of infection due to these strains (9,28–30). Surveillance data show that their prevalence is increasing in our capital cities, but the situation in rural Australia is not well documented (3). This increase will inevitably affect guidelines for empirical antimicrobial drug prescribing for staphylococcal infections and for patients in the community with suspected SAB. Further surveillance of staphylococcal infections, including bacteremia, is warranted to guide recommendations for empirical therapy and infection control interventions.
