# Correction: Vol. 11, No. 4

**DOI:** 10.3201/eid1111.C11110

**Published:** 2005-10

**Keywords:** Errata, Erratum, Correction, Vol. 11, No. 4

In “*Staphylococcus aureus Bacteremia*, Australia,” by Peter Collignon et al., an error occurred. On page 556, in Table 1, in line 10 of column 6, the value indicating total bloodstream infections over study period (all orgs) in Hospital E should be 1,296.

The corrected table appears in the online article at http://wwwnc.cdc.gov/eid/article/11/4/04-0772_article.htm

We regret any confusion this error may have caused.

